# The Therapeutic Effect and Clinical Outcome of Immune Checkpoint Inhibitors on Bone Metastasis in Advanced Non-Small-Cell Lung Cancer

**DOI:** 10.3389/fonc.2022.871675

**Published:** 2022-04-01

**Authors:** Yohei Asano, Norio Yamamoto, Satoru Demura, Katsuhiro Hayashi, Akihiko Takeuchi, Satoshi Kato, Shinji Miwa, Kentaro Igarashi, Takashi Higuchi, Hirotaka Yonezawa, Yoshihiro Araki, Sei Morinaga, Shiro Saito, Takashi Sone, Kazuo Kasahara, Hiroyuki Tsuchiya

**Affiliations:** ^1^ Department of Orthopedic Surgery, Kanazawa University Graduate School of Medical Sciences, Kanazawa, Japan; ^2^ Department of Respiratory Medicine, Kanazawa University Hospital, Kanazawa, Japan

**Keywords:** immune checkpoint inhibitor, non-small cell lung cancer, bone metastasis, bone modifying agent (BMA), denosumab

## Abstract

**Introduction:**

In advanced non-small-cell lung cancer (NSCLC), immune checkpoint inhibitors (ICIs) have been reported a better treatment outcome on primary lesions, however, the therapeutic effect on bone metastases has not been clarified. This study investigates the therapeutic effect of ICIs on bone metastases in advanced NSCLC.

**Methods:**

The data of patients with advanced NSCLC, treated with ICIs from 2016 to 2019 at our hospital, were analyzed. The therapeutic effects of ICIs on primary lung and metastatic bone lesions, concomitant use of bone modifying agents (BMA), treatment outcomes, and frequency of immune-related adverse events (irAEs) and skeletal-related events (SREs) were investigated.

**Results:**

A total of 29 patients were included (19 men and 10 women; mean age, 64.2 years). Among the ICIs, pembrolizumab was the most used (55.2%), and concomitant use of BMA was prevalent in 21 patients (zoledronic acid=1, denosumab=20). The therapeutic effect was partial response (PR) in 10.3% (n=3) on primary lung lesions by RECIST 1.1, complete response (CR) in 6.9% (n=2) and PR in 17.2% (n=5) on bone metastatic lesions by MDA criteria. ICIs suppressed the progression of bone metastasis in 21 cases (72.4%). All patients in CR and PR were treated with pembrolizumab and denosumab. SREs and irAEs were developed in 3.4% (n=1) and 20.7% (n=6), respectively. The median survival time after treatment with ICIs was 11.0 months. Concomitant therapy with ICIs and denosumab significantly prolonged the overall survival compared to ICI-only therapy (16.0 months vs. 2.5 months, p<0.01).

**Conclusions:**

This study showed that treatment with ICIs may successfully suppress the progression of bone metastasis in advanced NSCLC. Pembrolizumab with denosumab had the highest therapeutic effect on both primary lung lesions and bone metastases. Systemic treatment with this combination and conservative treatment of bone metastasis could be one of the options in the treatment of advanced NSCLC.

## Introduction

Lung cancer is a malignant neoplasm that is responsible for distant metastases at a relatively early stage in the disease, to the breast, prostate, and bones (1–3). It has been reported that 48% of patients with advanced (stage III, IV) non-small-cell lung cancer (NSCLC) have bone metastases at initial diagnosis (4). Bone metastases can cause skeletal-related events (SREs) such as severe bone pain, pathological fractures, spinal cord compression, and hypercalcemia (5), and can result in the deterioration of the quality of life (QOL). In advanced cases of lung cancer with bone metastasis, 20-30% of patients already have SREs at the time of diagnosis (4, 6, 7), and they have a worse prognosis than that of other cancers (8). SREs themselves could be independent predictors of worse prognosis in terms of overall survival (OS) (7), therefore, the treatment strategy for bone metastases is essential in NSCLC. A multidisciplinary approach with radiation therapy, surgical treatment, and bone modifying agents (BMAs) is necessary for the management of these cases. Zoledronic acid and denosumab have been reported to significantly reduce the incidence of SREs (9, 10) and prolong OS (11).

In recent years, it has been reported that immune checkpoint inhibitors (ICIs), which target the Programmed death-1 receptor (PD-1)/PD-1 ligand (PD-L1) pathway, have more favorable outcomes than conventional anticancer drugs on metastatic lung cancer (12–14). We have reported two cases of advanced NSCLC with a high risk of lower extremity long bone fracture due to metastases that were drastically improved by systemic treatment with pembrolizumab and denosumab (15). Two other case reports have shown the therapeutic effect of ICIs on bone metastasis in lung cancer (16, 17). Based on these findings, ICIs as well as their combination with BMAs such as denosumab may be one of the treatment options for NSCLC with bone metastases.

There is insufficient data regarding the therapeutic effect of ICIs on bone metastasis in NSCLC. The purpose of this study was to evaluate the therapeutic effect of ICIs on bone metastasis in advanced NSCLC and to seek a novel treatment strategy.

## Materials and Methods

### Study Design

This retrospective study analyzed patients diagnosed with advanced NSCLC and treated with ICIs between February 2016 and November 2019 in the Department of Respiratory Medicine of Kanazawa University Hospital, Japan. Patients diagnosed with bone metastases before the initiation of ICI treatment [PD-1 inhibitors (nivolumab and pembrolizumab) or PD-L1 inhibitor (atezolizumab)] and who received treatment for more than six months were included. Patients who died within six months were also included to assess the clinical outcomes of ICI treatment. On the other hand, patients who received concomitant therapy with a PD-1 inhibitor and Cytotoxic T-lymphocyte-associated protein 4 (CTLA-4) inhibitor were excluded. This study was approved by the ethics committee of the Kanazawa University Hospital (no. 3339-1) and has been carried out in accordance with the Declaration of Helsinki. Informed consent from the patients was obtained.

### Data Collection

The medical information used in this study was collected from the database at Kanazawa University Hospital. The collected data included patient demographics such as age and sex, disease details such as histological type, stage, Eastern Cooperative Oncology Group performance status (ECOG PS), metastatic sites, driver gene mutation, PD-L1 tumor proportion score (TPS), and treatment history. The therapeutic effects of ICIs on primary lung lesions and bone metastases were evaluated based on Response Evaluation Criteria in Solid Tumors (RECIST) version 1.1 (18) and MD Anderson Cancer Center (MDA) criteria (19), respectively. Change of the size of primary lung lesions and osteosclerotic change or tracer uptake of bone metastatic lesions were evaluated by computed tomography (CT) or bone scintigraphy. The evaluation at the final examination was investigated as the therapeutic effects of ICIs. In addition, concomitant use of BMAs such as zoledronic acid and denosumab, treatment outcomes, frequency of Immune-related adverse events (irAEs), and SREs were investigated.

### Statistical Analyses

Categorical variables were presented as frequencies and percentages, whereas continuous ones were presented as median (IQR). The Kaplan-Meier curve was used to evaluate the OS of the participants, and the log-rank test was performed for comparison amongst groups. The OS was defined as the period from the initiation of ICI treatment to death. A p-value < 0.05 was used to denote statistical significance. These analyses were performed using EZR (Saitama Medical Center, Jichi Medical University, Saitama, Japan) (20).

## Results

### Characteristics of Participants

A total of 29 patients (19 men and 10 women) were included in this study. The mean age was 64.2 years (44 – 81 years). The mean follow-up period after treatment with ICIs was 13.1 months (1 – 35 months). The most common histological type was adenocarcinoma in 75.9% (n = 22), and the most common disease stage was IVB (93.1%). The most common bone metastatic sites were the ribs (55.2%) and pelvis (55.2%), and most patients had multiple bone metastases (89.7%). In addition, metastases to other organs were observed in 75.9% (n = 22) of patients. The examination of driver gene mutation was performed in 25 patients and only one was positive for anaplastic lymphoma kinase mutation. The TPS, used for ICI treatment indication, was examined in 16 patients and 81.3% exhibited > 1% (n = 13) and 18.7% exhibited < 1% (n = 3). ICIs were the first-line therapy in 27.6% (n = 8), second-line in 44.8% (n =13), and third-line in 27.6% (n = 8) of the patients. Among them, eight patients who were treated with first-line ICIs had high PD-L1 TPS expression (≥ 50%). In the other patients who received ICI treatment as second-line or further treatment, surgery and radiation therapy for primary lung lesions were performed in 23.8% (n = 5) and 9.5% (n = 2), respectively, while anticancer drug treatment was performed in 100% (n = 21). None of the patients was treated with molecular-targeted drugs prior to ICI treatment. BMAs were administrated in 21 patients, 20 of whom received denosumab (69.0%), and one received zoledronic acid (3.4%) (**Table 1**).

**Table 1:** Characteristics of patients with advanced NSCLC treated with ICIs.

		Number (%)
Sex	Male	19 (65.5)
	Female	10 (34.5)
ECOG PS	0-2	26 (89.7)
	3-4	3 (10.3)
Histology	Adenocarcinoma	22 (75.9)
	Squamous cell carcinoma	3 (10.3)
	Pulmonary pleomorphic carcinoma	2 (6.9)
	Poor-differentiated carcinoma	2 (6.9)
Driver gene mutation: (positive/total)	EGFR	0/25
	ALK	1/24
	ROS1	0/8
	Untested	4
Number of bone metastasis	Solitary	3 (10.3)
	Multiple	26 (89.7)
Site of bone metastasis	Ribs	16 (55.2)
	Pelvis	16 (55.2)
	Thoracic spine	15 (51.7)
	Lumbar spine	11 (37.9)
	Long bone of the extremities	5 (17.2)
	Scapula	5 (17.2)
	Other	6 (20.7)
Metastasis to other organs	Yes	22 (75.9)
	No	7 (24.1)
PD-L1 TPS	≥50%	9 (36.0)
	1-49%	4 (16.0)
	<1%	3 (12.0)
	Untested	13 (52.0)
ICI	Nivolumab	11 (37.9)
	Pembrolizumab	16 (55.2)
	Atezolizumab	2 (6.9)
Therapy line	1st	8 (27.6)
	2nd	13 (44.8)
	≥3rd	8 (27.6)
Treatment history prior to ICI treatment	Surgery of primary lung lesions	5 (23.8)
	Radiation therapy of primary lung lesions	2 (9.5)
	Anticancer drug	21 (100)
	Molecular-targeted drug	0
BMA	Denosumab	20 (69.0)
	Zoledronic acid	1 (3.4)

ECOG PS, Eastern Cooperative Oncology Group Performance Status; EGFR, epidermal growth factor receptor; ALK anaplastic lymphoma kinase; PD-L1 programmed death-ligand 1; TPS, tumor proportion score; ICI, immune checkpoint inhibitor; BMA, bone modifying agent.

### Therapeutic Effects

**Figure 1:**
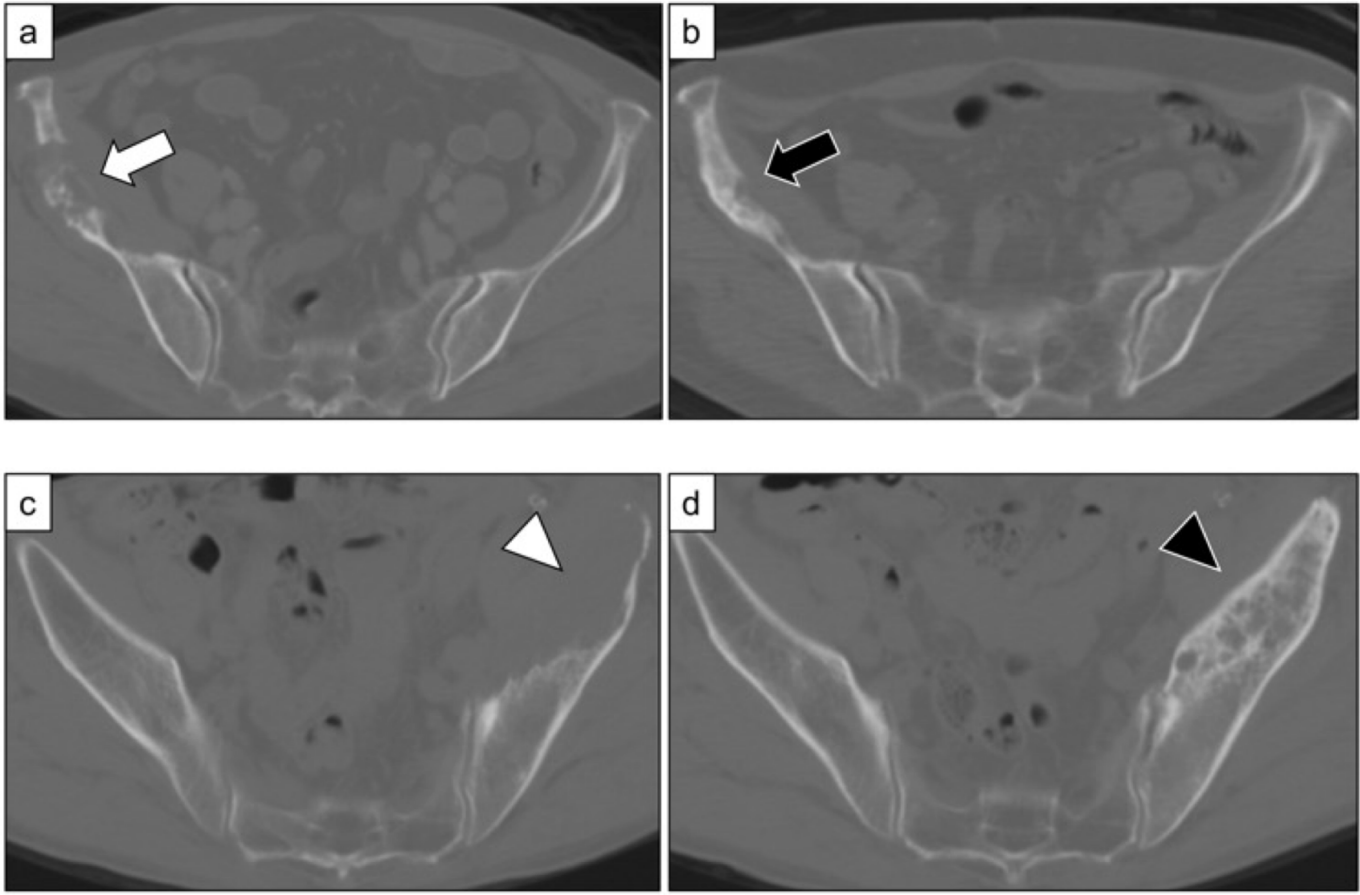
**(A)** Metastatic bone lesions in the right ilium of a 77-year-old woman (Case 17). **(B)** Osteosclerotic changes three months after treatment with pembrolizumab and denosumab. The patient was in partial remission (MDA criteria). **(C)** Metastatic bone lesions in the left ilium of a 64-year-old man (Case 29). **(D)** Osteosclerotic changes five months after treatment with pembrolizumab and denosumab. The patient was in partial remission (MDA criteria).

Treatment with ICIs suppressed the progression of bone metastasis in 21 out of the 29 cases (72.4%). The most commonly used ICI was pembrolizumab (55.2%, n = 16), which had the best therapeutic effect on both primary lung lesions (partial response (PR) in 18.8% (n = 3) according to RECIST 1.1) and bone metastases where osteosclerotic changes were noted, indicating a response to therapy (complete response [CR] in 12.5% [n = 2] and PR in 31.2% [n = 5] according to the MDA criteria) (**Table 2** and **Figure 1**). Nivolumab and atezolizumab on lung lesions and bone metastases resulted in either stable disease (SD) or progressive disease (PD) in all patients (**Table 2**).

**Table 2:** Therapeutic effect of ICIs on primary lung lesions and bone metastases.

	Nivolumab	Pembrolizumab	Atezolizumab	Total
	N = 11 (37.9%)	N = 16 (55.2%)	N = 2 (6.9%)	N = 29
Therapy line: N (%)				
1st	0 (0)	8 (50.0)	0 (0)	8 (27.6)
2nd	7 (63.7)	5 (31.2)	1 (50.0)	13 (48.3)
≧3rd	4 (33.3)	3 (18.8)	1 (50.0)	8 (24.1)
RECIST 1.1: N (%)				
CR	0 (0)	0 (0)	0 (0)	0 (0)
PR	0 (0)	3 (18.8)	0 (0)	3 (10.3)
SD	2 (18.2)	3 (18.8)	0 (0)	5 (17.2)
PD	8 (72.7)	9 (56.2)	2 (100)	19 (65.5)
N/A	1 (9.1)	1 (6.2)	0 (0)	2 (7.0)
MDA criteria: N (%)				
CR	0 (0)	2 (12.5)	0 (0)	2 (7.0)
PR	0 (0)	5 (31.2)	0 (0)	5 (17.2)
SD	5 (45.4)	7 (43.8)	2 (100)	14 (48.3)
PD	5 (45.4)	2 (12.5)	0 (0)	7 (24.1)
N/A	1 (9.1)	0 (0)	0 (0)	1 (3.4)

ICI, immune checkpoint inhibitor; RECIST 1.1, Response Evaluation Criteria in Solid Tumors version 1.1; MDA criteria, MD Anderson Cancer Center criteria; CR, complete response; PR, partial response; SD, stable disease; PD, progressive disease; N/A, not available.

All patients whose evaluations showed CR or PR with regard to bone metastases were treated with pembrolizumab as the first- or second-line therapy and received concomitant use of denosumab. Moreover, three patients had PR in terms of primary lung lesions (**Table 3**). In these patients, denosumab was initiated at the same time as ICI treatment compared with those who were evaluated as SD or PD (71.4% vs. 0%, p<0.01). However, there was no significant difference in the duration of concomitant use of ICIs and denosumab (CR or PR; 7.9 months vs. SD or PD; 7.5 months, p=0.92).

**Table 3:** Therapeutic effect of ICI and DEMb concomitant therapy compared to ICI-only or ICI and ZOL therapy.

	ICI only (n = 8) or ICI + ZOL (n = 1)	ICI + DMAb (N = 20)		
		Nivolumab	Pembrolizumab	Atezolizumab
		(N = 10)	(N = 9)	(N = 1)
RECIST 1.1				
CR	0	0	0	0
PR	0	0	3	0
SD	2	1	2	0
PD	6	8	4	1
N/A	1	1	0	0
MDA criteria				
CR	0	0	2	0
PR	0	0	5	0
SD	7	4	2	1
PD	2	5	0	0
N/A	0	1	0	0

ICI, immune checkpoint inhibitor; DMAb, Denosumab; ZOL, Zolendronic acid; RECIST 1.1, Response Evaluation Criteria in Solid Tumors version 1.1; MDA criteria, MD Anderson Cancer Center criteria; CR, complete response; PR, partial response; SD, stable disease; PD, progressive disease; N/A, not available.

### Prognosis

**Figure 2:**
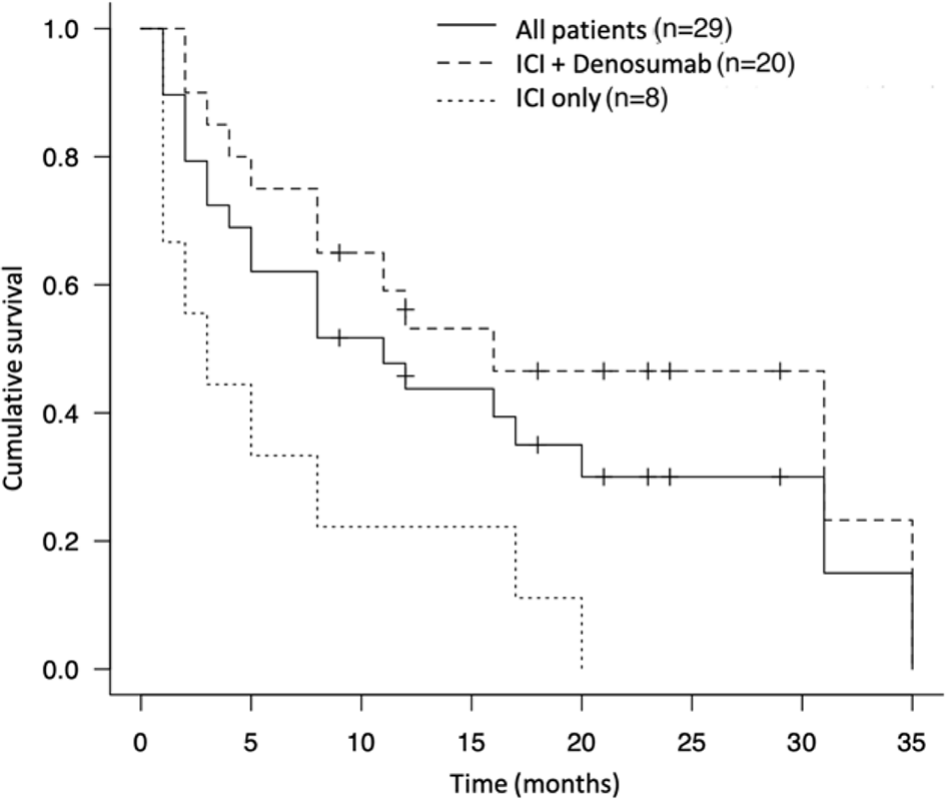
Kaplan-Meier survival curve of the therapeutic effect of immune checkpoint inhibitor and denosumab and immune checkpoint inhibitor alone.

**Figure 3:**
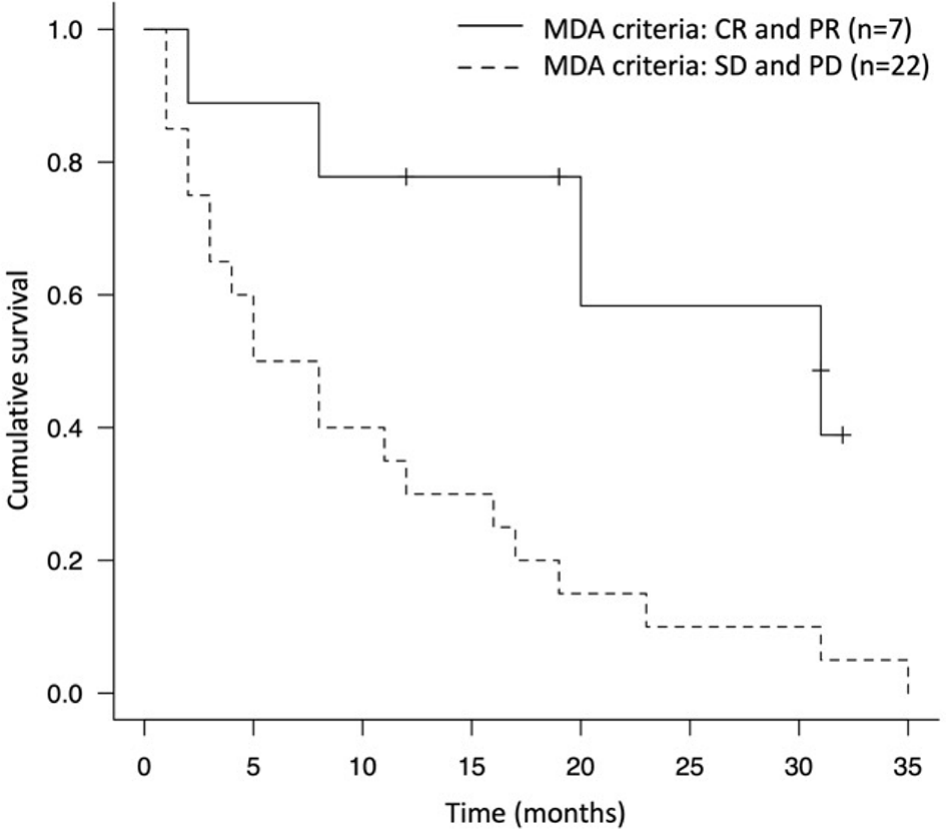
Kaplan-Meier survival curve of the group that immune checkpoint inhibitor responded to bone metastatic lesions (MDA criteria evaluation was CR and PR) and the non-responded group (MDA criteria evaluation was SD and PD).

During the study period, the median survival time after treatment with ICIs was 11.0 months (4 – 20 months) and 72.4% of the patients died from the disease (n = 21) (**Table 4**). The 1-year and 2-year survival rates were 44.8% and 24.0%, respectively. Concomitant therapy with ICIs and denosumab significantly prolonged the OS compared to ICI-only therapy (16.0 months vs. 2.5 months, p<0.005) (**Figure 2**). In the concomitant therapy and ICI-only therapy groups, ICIs were used as more than third-line therapy in 25% (n = 5) and 25% (n = 2) of patients, respectively, and there was no significant difference (p=0.98). In addition, there were no significant differences in the type of ICI used for treatment (p=0.25) or in the ECOG PS before ICI treatment (p=0.35) between the groups. Moreover, the OS of the group that ICIs responded to bone metastatic lesions was significantly prolonged than that of the non-responsive group (19.3 months vs. 10.3 months, p=0.0072) (**Figure 3**).

**Table 4:** Patient characteristics, disease features, treatment details, and outcomes of study population.

Case/Age/Sex	Histology	Number of bone metastasis	PD-L1	ICI	MDA criteria	RECIST 1.1	BMA	irAEs	Outcome
			TPS (%)					(Grade)	
1/67/M	SQCC	solitary	N/T	NIV	SD	PD	DMAb	No	DOD
2/77/F	ADC	multiple	N/T	NIV	PD	N/A	DMAb	No	DOD
3/47/F	ADC	multiple	N/T	NIV	PD	PD	DMAb	No	DOD
4/54/M	ADC	multiple	N/T	NIV	PD	PD	DMAb	No	DOD
5/59/M	PPC	multiple	N/T	NIV	SD	PD	DMAb	Encephalitis (G5)	DOD
6/57/M	P/D	multiple	N/T	NIV	SD	SD	DMAb	No	AWD
7/60/M	PPC	multiple	N/T	NIV	SD	SD	No	No	DOD
8/66/M	ADC	multiple	N/T	NIV	PD	PD	DMAb	No	DOD
9/61/M	ADC	solitary	N/T	NIV	N/A	PD	DMAb	No	DOD
10/67/M	ADC	multiple	N/T	NIV	PD	PD	DMAb	Hypothyroidism (G2)	DOD
11/71/M	SQCC	solitary	N/T	PEM	PD	PD	ZOL	No	DOD
12/60/M	ADC	multiple	25-49	PEM	SD	PD	No	No	DOD
13/62/M	ADC	multiple	<1	NIV	SD	PD	DMAb	No	AWD
14/74/F	ADC	multiple	75	PEM	CR	PR	DMAb	No	AWD
15/68/F	ADC	multiple	>75	PEM	SD	SD	DMAb	No	DOD
16/46/M	ADC	multiple	N/T	PEM	SD	PD	No	No	DOD
17/77/F	ADC	multiple	>75	PEM	PR	PD	DMAb	Pneumonitis (G2)	AWD
18/66/F	ADC	multiple	100	PEM	SD	N/A	No	No	DOD
19/81/M	ADC	multiple	>75	PEM	SD	PD	DMAb	Drug eruption (G2)	DOD
20/64/M	ADC	multiple	80-90	PEM	SD	SD	No	No	DOD
21/46/F	ADC	multiple	11-24	PEM	PR	PD	DMAb	No	DOD
22/75/F	ADC	multiple	>75	PEM	PR	SD	DMAb	Cholangitis (G2)	DOD
23/73/M	SQCC	multiple	25-49	PEM	SD	PD	No	No	DOD
24/69/M	ADC	multiple	24	PEM	PD	PD	No	No	DOD
25/44/F	P/D	multiple	70-80	PEM	PR	PD	DMAb	No	AWD
26/69/F	ADC	multiple	<1	ATE	SD	PD	No	No	DOD
27/66/M	ADC	multiple	N/T	ATE	SD	PD	DMAb	Hypothyroidism (G2)	AWD
28/71/M	ADC	multiple	>75	PEM	CR	PR	DMAb	No	AWD
29/64/M	ADC	multiple	<1	PEM	PR	PR	DMAb	No	AWD

ICI, immune checkpoint inhibitor; ADC, adenocarcinoma; SQCC, squamous cell carcinoma; PPC, pulmonary pleomorphic carcinoma; P/D poor-differentiated carcinoma; N/T, not tested; RECIST 1.1, Response Evaluation Criteria in Solid Tumors version 1.1; MDA criteria, MD Anderson Cancer Center criteria; CR, complete response; PR, partial response; SD, stable disease; PD, progressive disease; N/A, not available; DMAb, Denosumab; ZOL, Zolendronic acid; DOD, dead of disease; AWD, alive with disease.

### Adverse Events

irAEs were observed in 20.7% (n = 6) and were encephalitis (Common Terminology Criteria for Adverse Events (CTCAE) grade 5), pneumonitis (grade 2), cholangitis (grade 2), drug eruption (grade 2) and hypothyroidism (grade 2). One patient who developed encephalitis and died was treated with nivolumab and developed irAEs after two cycles of treatment; magnetic resonance imaging (MRI) revealed abnormal lesions in the bilateral temporal lobes. Based on these results, nivolumab-induced autoimmune encephalitis was diagnosed clinically. In contrast, the other five patients with irAEs were stable after ICI discontinuation and steroid treatment. Among those who died, nivolumab was used in two patients, pembrolizumab in three, and atezolizumab in one (**Table 4**). Only one patient developed SRE (spinal cord compression at the level of the 5^th^ thoracic spine) after the initiation of nivolumab and was treated by radiation therapy.

## Discussion

In this study, the bone metastasis control rate of advanced NSCLC treated with ICIs was 72.4%, two of those patients were in CR and five were in PR based on MDA criteria. All patients received pembrolizumab and denosumab, implying that the combined treatment may have high therapeutic effects on bone metastases in advanced NSCLC.

In recent years, immunotherapy, including ICIs, has made remarkable progress and led to a paradigm shift in cancer treatment (21). Some reports have shown that ICIs prolong OS and progression-free survival (PFS) in patients with metastatic NSCLC compared with conventional anticancer medications (12–14), and they are expected to improve the prognosis and QOL of these patients. However, in advanced NSCLC, there are few reports on the therapeutic effects of ICIs on bone metastases. Therefore, this study examined the efficacy of ICIs on bone metastases in advanced NSCLC.

In this study, seven patients (24.1%) had osteosclerotic changes of bone metastases after the initiation of ICIs, showing response to therapy (CR in two patients and PR in five patients). All these patients had been treated with pembrolizumab as first-line therapy in six patients and second-line in one patient. The two patients with CR of bone metastases were in PR in terms of the primary lung lesions (15). Similarly, two case reports showed the remarkable effectiveness of pembrolizumab on both primary lung lesions and bone metastases (16, 17). In both these case reports, pembrolizumab was used as first-line therapy, and the bone metastases disappeared. Moreover, the seven patients who showed improvement in the osteosclerotic changes of bone metastases had been received the combined treatment of ICIs and denosumab. In NSCLC, although denosumab has been reported to prolong OS and prevent SREs (9, 11, 22), there have been no reports on the correlation between osteosclerotic changes in osteolytic lesions of bone metastases, including impending fractures, and ICIs. Therefore, it was considered that denosumab may enhance the therapeutic effects of ICIs. There are only a few reports regarding the therapeutic effect of ICIs on the primary lung lesions treated with the combined therapy of ICIs and denosumab for NSCLC (23), but none have investigated the therapeutic effect on bone metastases. Thus, the correlation between these drugs has not been clarified. In previous case reports where metastatic bone lesions disappeared after treatment with pembrolizumab, BMAs such as denosumab were not used (16, 17). Our results show that among ICIs, pembrolizumab may have the highest therapeutic effects both on primary lung lesions and bone metastases.

The combined effect of ICIs and denosumab have been previously studied in other malignancies. Angela et al. reported that the combined treatment of PD-1 inhibitor and denosumab resulted in osteosclerotic changes on bone metastases in 62% of stage IV melanoma (24). Furthermore, in a clinical study, a longer mean duration of concomitant use of ICIs and denosumab was associated with increased overall response rate (ORR) (CR + PR) in melanoma and NSCLC as well as increased overall survival in NSCLC (23). On the other hand, Qin et al. reported that the use of BMA was not associated with decreased SREs or differences in survival in patients with metastatic NSCLC (25). Thus, the influence of the concomitant use of denosumab in ICI treatment on clinical outcomes has not yet been clarified, and the prognostic significance of ICI treatment in NSCLC with bone metastases remains inconsistent (26).

It has been shown that Receptor activator of nuclear factor kappa-B ligand (RANKL) inhibitors improve the anti-metastatic activity of PD-1/PD-L1 inhibitors, resulting in subcutaneous growth suppression in mouse models of melanoma, prostate cancer, and colon cancer (27). Additionally, the RANKL/RANK axis has been shown to affect the immune response such as regulatory, CD8+ and CD4+ T cells, as well as myeloid suppressor cells (28–32). Ahern et al. reported that optimal antitumor effects were observed when anti-RANKL was initiated concurrently with or after ICI treatment (27). In our study, denosumab was initiated at the same time as ICI treatment in cases where the therapeutic effect on bone metastasis was CR or PR. These reports support the results of our study, except for the duration of concomitant use of denosumab. Regarding that duration, our results showed no significant difference in the response of ICIs to bone metastasis. However, five out the seven patients evaluated as CR or PR based on the MDA criteria were still treated with combined therapy, which may make a significant difference in the future. The exact mechanism underlying the antitumor effect of the concomitant use of ICIs and denosumab requires further analysis.

In this study, TPS was evaluated in six out the seven patients who had CR and PR in terms of bone metastases, and high expression was observed in five patients, but only one patient exhibited < 1%. TPS is measured before the initiation of pembrolizumab in unresectable advanced or recurrent NSCLC. If it is > 1%, first-line treatment with pembrolizumab alone is possible (14). High expression of PD-L1 in NSCLC is a predictive factor of the therapeutic effectiveness of pembrolizumab on primary lung lesions (33, 34), however, there are no reports regarding the effect on bone metastases. The results of this study implied that the high TPS expression might be a predictive factor of high therapeutic effect on both bone metastases and primary lung lesions.

The irAEs of ICIs are other important issues that need to be considered. Sun et al. reported that the overall incidence of irAEs in NSCLC was 22% for all grades and 4% for high grades in a systematic review and meta-analysis (35). In our study, 20.7% (n = 6) developed irAEs and 3.4% (n = 1) of those who developed encephalitis died (grade 5), consistent with the report. From these patients, two had achieved PR and the others had SD on bone metastasis evaluation. In NSCLC patients who were treated with ICIs, it has been reported that PFS, OS, and ORR of the patients who developed irAEs were more favorable than those who did not (36–39). However, there have been no studies regarding the therapeutic effect association with irAEs and bone metastases. Our results show that the incidence of irAEs might correlate with the therapeutic effect, and further studies are necessary to clarify these correlations.

In our study, three patients had impending fractures of the long bone of the lower extremity, two of them who were in CR based on the MDA criteria (15) and had Mirels scores (40) of 9 and 11 points. Since they were simultaneously diagnosed with primary lung cancer and bone metastasis, systemic treatment with pembrolizumab was prioritized, and conservative treatment was decided upon regarding the bone metastases after multidisciplinary discussions. The bone remodeling of the metastatic lesions was observed during the follow-up period, and they were able to return to their normal daily life without prophylactic surgery for impending fractures. One of the patients with SD in terms of bone metastasis was prioritized systemic treatment with pembrolizumab due to brain metastasis. Since the bone metastasis progression was suppressed by the systemic treatment, prophylactic surgery was not necessary. In addition, three other patients had extremity long bone metastases, the proximal humerus in one patient and the femoral trochanter in two patients. The Mirels scores of these patients were < 8 points for the proximal humerus, but 9 points for the patient with the femoral trochanter metastasis.

Prophylactic surgery for impending fractures of the lower extremities is a reasonable approach (41, 42), however, it is not always beneficial for advanced-stage patients due to poor general health and systemic treatment delays. This study revealed two cases of impending fractures that responded to ICIs and one whose progression was suppressed, and thus avoided prophylactic surgery. These results suggested that the systemic treatment of pembrolizumab and denosumab with conservative treatment with regards to bone metastasis could be one of the options for advanced NSCLC, although further studies are needed.

There were some limitations in this study. The number of patients was small. In addition, no control group received medication therapy other than ICIs, and the therapeutic effects of ICIs could not be compared with the effects of these therapies. Moreover, the effects of chemotherapy administered concurrently with ICIs were not considered. Although the concomitant use of denosumab in ICI treatment might enhance the therapeutic effect, the mechanism has not been sufficiently elucidated, and further verification of this finding is required.

In conclusion, ICIs suppressed the progression of bone metastasis in advanced NSCLC in 72.4% of this limited case series, although most cases were combined with BMAs. Pembrolizumab with denosumab had the highest therapeutic effect on not only primary lung lesions but bone metastases as well. These results suggest that the systemic treatment of pembrolizumab with denosumab with conservative treatment of bone metastasis could be one of the options for advanced NSCLC even in cases of impending lower extremity fracture. Future research should focus on validating these results, as well as creating guidelines to manage the bone metastasis from advanced NSCLC with a multidisciplinary approach including ICIs, anticancer drugs, molecular-targeted drugs, BMAs, surgical intervention, and radiotherapy.

## Data Availability Statement

The original contributions presented in the study are included in the article/supplementary material. Further inquiries can be directed to the corresponding author.

## Ethics Statement

Written informed consent was obtained from the individual(s) for the publication of any potentially identifiable images or data included in this article.

## Author Contributions

The manuscript was drafted by YoA, and AT. YoA, SD, KH, AT, SK, ShM, KI, TH, HY, YA, SeM, SS, and TS collected and analyzed the data and YoA wrote the manuscript. NY, AT, KK, and HT revised the manuscript. All authors were involved in the preparation of this study, and read and approved the final manuscript.

## Conflict of Interest

The authors declare that the research was conducted in the absence of any commercial or financial relationships that could be construed as a potential conflict of interest.

## Publisher’s Note

All claims expressed in this article are solely those of the authors and do not necessarily represent those of their affiliated organizations, or those of the publisher, the editors and the reviewers. Any product that may be evaluated in this article, or claim that may be made by its manufacturer, is not guaranteed or endorsed by the publisher.
